# The Measurement of Dose and Response for Smoking Behavior Change Interventions in the Digital Age: Systematic Review

**DOI:** 10.2196/38470

**Published:** 2022-08-25

**Authors:** Megumi Ichimiya, Raquel Gerard, Sarah Mills, Alexa Brodsky, Jennifer Cantrell, W Douglas Evans

**Affiliations:** 1 Department of Prevention and Community Health Milken Institute School of Public Health The George Washington University Washington, DC United States; 2 Schroeder Institute Truth Initiative Washington, DC United States; 3 Department of Social and Behavioral Sciences School of Global Public Health New York University New York, NY United States

**Keywords:** digital health, digital media, social media, behavior change interventions, smoking, vaping, dose-response, telehealth, mobile health, mHealth, mobile phone

## Abstract

**Background:**

There is little consensus regarding effective digital health interventions for diverse populations, which is due in part to the difficulty of quantifying the impact of various media and content and the lack of consensus on evaluating dosage and outcomes. In particular, digital smoking behavior change intervention is an area where consistency of measurement has been a challenge because of emerging products and rapid policy changes. This study reviewed the contents and outcomes of digital smoking interventions and the consistency of reporting to inform future research.

**Objective:**

This study aims to systematically review digital smoking behavior change interventions and evaluate the consistency in measuring and reporting intervention contents, channels, and dose and response outcomes.

**Methods:**

PubMed, Embase, Scopus, PsycINFO, and PAIS databases were used to search the literature between January and May 2021. General and journal-based searches were combined. All records were imported into Covidence systematic review software (Veritas Health Innovation) and duplicates were removed. Titles and abstracts were screened by 4 trained reviewers to identify eligible full-text literature. The data synthesis scheme was designed based on the concept that exposure to digital interventions can be divided into intended doses that were planned by the intervention and enacted doses that were completed by participants. The intended dose comprised the frequency and length of the interventions, and the enacted dose was assessed as the engagement. Response measures were assessed for behaviors, intentions, and psychosocial outcomes. Measurements of the dose-response relationship were reviewed for all studies.

**Results:**

A total of 2916 articles were identified through a database search. Of these 2916 articles, the title and abstract review yielded 324 (11.11%) articles for possible eligibility, and 19 (0.65%) articles on digital smoking behavior change interventions were ultimately included for data extraction and synthesis. The analysis revealed a lack of prevention studies (0/19, 0%) and dose-response studies (3/19, 16%). Of the 19 studies, 6 (32%) reported multiple behavioral measures, and 5 (23%) reported multiple psychosocial measures as outcomes. For dosage measures, 37% (7/19) of studies used frequency of exposure, and 21% (4/19) of studies mentioned the length of exposure. The assessment of clarity of reporting revealed that the duration of intervention and data collection tended to be reported vaguely in the literature.

**Conclusions:**

This review revealed a lack of studies assessing the effects of digital media interventions on smoking outcomes. Data synthesis showed that measurement and reporting were inconsistent across studies, illustrating current challenges in this field. Although most studies focused on reporting outcomes, the measurement of exposure, including intended and enacted doses, was unclear in a large proportion of studies. Clear and consistent reporting of both outcomes and exposures is needed to develop further evidence in intervention research on digital smoking behavior change.

## Introduction

### Background

There are few published data on exposure to and evaluations of digital behavior change interventions, and to date, there is little consensus regarding effective interventions for diverse populations. This is because of the difficulty of quantifying digital health interventions that use various media and content and the lack of consensus on how to evaluate and report dose and outcomes. However, digital health is developing rapidly, and understanding the latest evidence is critical for research in this field.

Digital media is central to and an integral part of modern life; however, the study of its effects on health behavior is just beginning [1]. As reported by the World Bank, 45% of the world’s population or 3.5 billion people use social media. Worldwide, the average user spends approximately 3 hours of their day on social media [2]. Given the widespread exposure to digital technologies such as social media, it is increasingly important to understand how digital media affects individual health decision-making and behavior, as well as social networks and communities. These facts make it critically important to understand how digital media influences behavior. Social and behavioral scientists who study digital media must learn how to design and evaluate effective behavior change interventions, the evidence-based approaches that are effective, and how digital media affects targeted outcomes.

Efforts to understand the practice of digital media interventions have historically been made by public health scientists. Hu [3] reviewed 348 journal articles and structured the subject, health topics, technologies, and methods used for digital interventions between 2008 and 2012. Abad et al [4] conducted a scoping review on digital public health surveillance and revealed that only 0.8% of the related studies between 2005 and 2020 deployed a digital health surveillance system that can be used for monitoring and targeted interventions, despite its impact on the study methodology and public health actions. A recent systematic review by Seiler et al [1] found relatively few rigorous studies on the effectiveness of digital media–related behavior change campaigns and interventions. This review also found that the reporting of design, measures, data collection, and other methods needs to be improved and systematized. Recommendations for improvements included clarification of what is meant by *dose* and *dose-response*; how and with what intensity interventions are delivered; and measurement of outcomes, including attitudes, beliefs, social norms, and health behavior. This review also reiterated a previous finding that evidence for behavior change using digital interventions stems primarily from studies conducted in high-income countries [5-9].

Although digital media has been used for a variety of public health programs, one of the most rapidly changing areas is digital smoking behavior change interventions. Owing to the emerging products, devices, and policy changes, it has been more difficult to systematically quantify and evaluate the effectiveness of interventions in this area. Despite the importance of using consistent measures and evaluation methods to understand the impacts of interventions, little effort has been made to understand the common measures and methods used to evaluate the effectiveness of these interventions. There is a need for an assessment of the measures used in this area to inform future research to accurately evaluate the outcomes across a variety of products and devices, as well as rapid policy and market change. To the authors’ knowledge, no previous systematic review has focused on the detailed measurements used in digital smoking interventions.

Although tobacco use has declined overall [10], it remains at unacceptable levels, exacting personal and social costs, particularly among young adults [11,12]. Tobacco is the leading preventable cause of death in the United States [13]. In the United States, 18.2% of young adults aged 18 to 24 years reported current use of tobacco products, and 10.4% reported being current cigarette smokers [14]. Although the age at which smokers initiate cigarette use has been increasing over time, almost all cigarette use initiation occurs before the age of 26 years [12], making young adults a critical target for prevention efforts. Given the widespread use of digital media among the young adult population [15], digital interventions may be promising.

### Objective

To address these gaps in digital smoking behavior change interventions, this review aimed to (1) systematically review and codify the measures used for digital health interventions in tobacco and nicotine use research, (2) evaluate the quality of reporting of dose and response, and (3) identify areas for improvement in the field.

## Methods

This review followed the PRISMA (Preferred Reporting Items for Systematic Reviews and Meta-Analyses) guidelines. The protocol was registered in PROSPERO (International Prospective Register of Systematic Reviews; ID CRD42021285655).

### Study Search

The PubMed, Embase, Scopus, PsycINFO, and PAIS databases were used to search the literature. The search terms included “digital intervention,” “health promotion,” “health education,” “health communication,” “digital technology,” “social media,” “social marketing,” “health,” “measures,” “methods,” “frequency,” “impression,” and “reach.” General and journal-based searches were combined to find literature that was directly related to the scope of the study. Specific journals that were searched included *The Lancet Digital Health*, *Journal of Medical Internet Research*, *Digital Health*, *Social Networks*, *npj Digital Medicine*, *Digital Medicine*, *Digital Biomarkers*, *Frontiers in Digital Health*, *Communication Methods and Measures*, *Health Technology Assessment*, *BMC Medical Research Methodology*, *Computers in Human Behavior*, *Computers in Biology and Medicine*, *Journal of Health Communication*, *Journal of Communication in Healthcare*, *Health Communication*, *Health Communication Science Digest*, *Health Education and Behavior*, *Digital Medicine*, *International Journal of Digital Healthcare*, *Journal of Health and Social Behavior*, *American Journal of Health Behavior*, *Journal of Behavioral Health*, *The Journal of Behavioral Health Services and Research*, *Health Behavior Research*, *American Journal of Health Promotion*, *Health Promotion Practice*, *Journal of Prevention and Health Promotion*, *Health Promotion International*, *International Journal of Health Promotion and Education*, and *Journal of Health Promotion and Behavior*. The literature was searched between January and May 2021, followed by a series of monthly searches to identify additional studies. Search strategies and terms were developed in collaboration with librarians at George Washington University.

All records searched through the database were imported into Covidence systematic review software (Veritas Health Innovation) and duplicates were removed.

### Screening

Titles and abstracts were screened by 4 trained reviewers to identify eligible full-text literature. Each study was screened by 2 reviewers, and disagreements were resolved through discussion between the reviewers. The inclusion criteria were (1) publication after 2000, (2) full text available in English, (3) peer-reviewed original journal articles, (4) health-related topics, (5) at least one behavior change intervention defined, (6) use of the internet or mobile-based platform for mass or targeted communication, (7) use of digital devices, and (8) measurement of original data related to behavior or psychosocial measures.

Behavior change interventions were defined as planned programs that had stated objectives related to behavior change, target populations, and targeted messages in text, audio, video, graphics, or other distributed forms in a one-to-many format. Studies that assessed at least one behavioral or psychosocial measure defined in the social behavioral theories were included. Studies that only measured engagement were excluded. Digital media is defined as an internet-based platform for mass and targeted communications, including social media, apps, websites, software, blogging, and one-to-one chat platforms used for mass and targeted communications (eg, WhatsApp). Video games, emails, radio, and television were excluded from digital media. Studies that used digital media as a channel for one-to-one communication such as conversations between health care providers and patients were also excluded.

After literature was screened using the abovementioned criteria, articles related to smoking were extracted for this review. The terms used for this process included “tobacco,” “smoking,” “cigarette,” “vaping,” “vape,” “e-cig,” “ENDS,” “nicotine,” “hookah,” “JUUL,” “cigar,” “e-liquid,” “flavor,” “smokeless,” “smoker,” and “vaper.” The literature search was repeated replacing the term “health” with “smoking” and “vaping” on the database, and we checked for the coverage of the articles on the abovementioned topics.

### Data Extraction and Synthesis

The data extraction and synthesis scheme was designed to identify the format of the digital media intervention, the measurement of each component that assessed dose and response in the intervention, and the study design used in the research. Dose measurement items were developed based on the concept that exposure to digital health interventions can be divided into 2 parts [16]. The first part is the intended dose, which refers to planned exposure by the intervention side. The other part is an enacted dose that corresponds to a portion of the intended dose that is actually completed by the participant. The dose measurement items were designed so that doses can be expressed as the frequency of the intervention multiplied by the length of intervention component and amount of engagement, which offers supplemental information about active involvement by participants. In this review, the intended dose comprises the frequency and length of the intervention, and the enacted dose comprises engagement. Engagement was defined as the interaction between the intervention content and the participants, such as views, clicks, likes, comments, and shares. Response equals the outcomes of the intervention, including behavior, intention, and other psychosocial factors that were previously confirmed to be connected in social behavioral change theories. A dose-response relationship was defined as the association between different levels of doses (exposure) and responses (outcomes), and its application was assessed in this review.

The codebook for data extraction and synthesis was developed in a Microsoft Excel format and piloted using 20 randomly selected articles on digital health behavior change interventions. The extracted data included basic information about the study, types of digital media and devices used, modes of intervention, measures used for intervention exposure, outcomes, engagement, study designs, model applications, cost and funding information, and source of bias. Data were converted into standardized forms where necessary and checked for clarity of reporting. The codebook had 3 types of input formats, including categorical options with data validation; hence, coders could only type prespecified responses, dichotomous options with data validation that indicated yes or no, and free answers where coders could leave notes. Coders were the same as the reviewers in the screening phase, who had 4 weeks of training using this codebook. The coding for this review started after the interrater reliability met 80% agreement in the training. Disagreements were resolved by discussion. The specific items extracted and synthesized are summarized in the *Results* section.

## Results

### Overview

**Figure 1:**
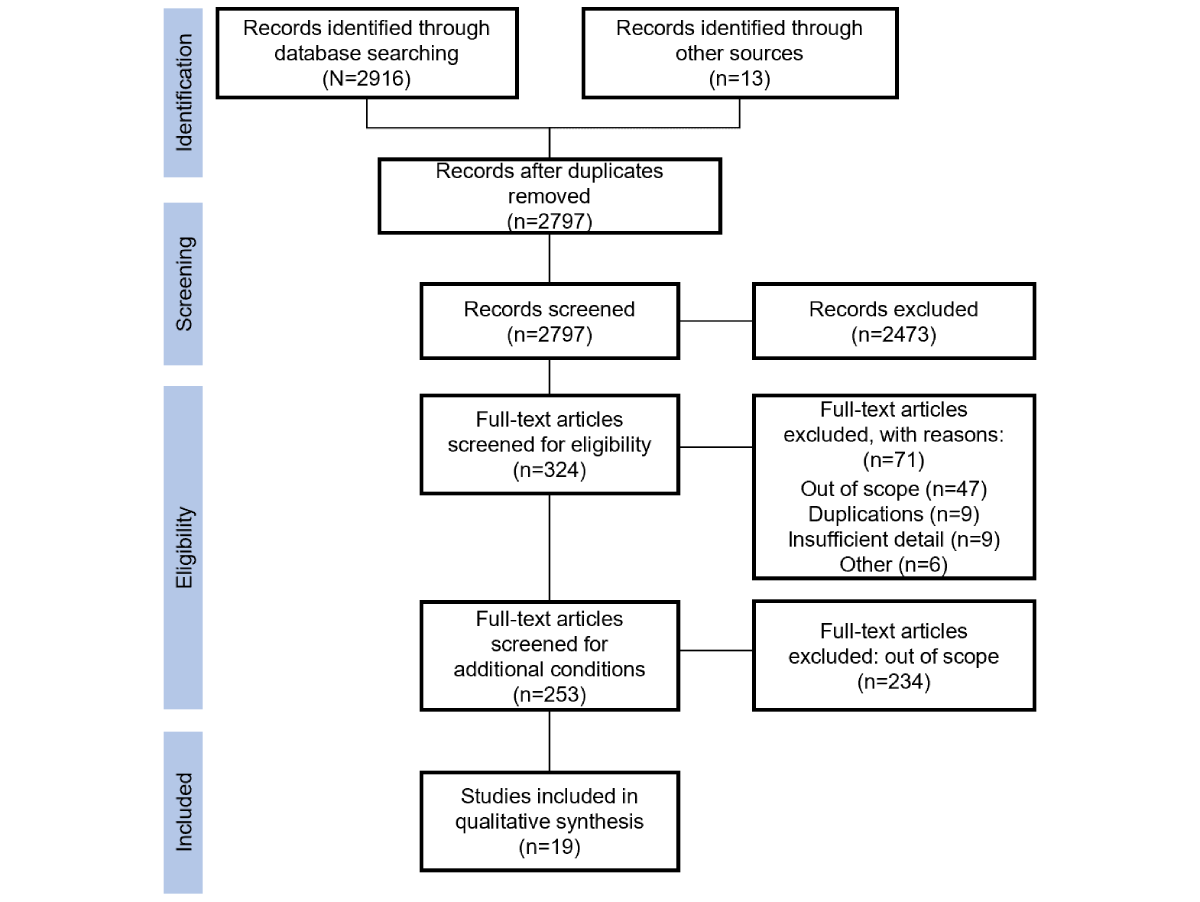
Flowchart of literature screening.

A total of 2916 articles were identified through a database search, of which 2797 (95.92%) records were screened for titles and abstracts after duplicates were removed. Title and abstract review of the 2797 articles yielded 324 (11.58%) articles for possible eligibility, and 253 (9.05%) articles were screened for the subject matter. Ultimately, of the 253 articles, 19 (7.5%) articles on digital smoking behavior change interventions were included for data extraction and synthesis. The flow of literature screening is presented in Figure 1.

### Digital Interventions

A summary of digital smoking behavior change interventions is presented in Table 1. Of the 19 studies included, 13 (68%) focused on smoking cessation and some also included smoking reduction as a secondary outcome that led to smoking cessation. Several articles focused on promoting a social movement for rejecting tobacco and reducing the influence of peer smoking, and others had more general topics such as promoting healthy lifestyles, tobacco-free lifestyles, and antitobacco norms.

Of the 19 studies, 10 (53%) studies used multiple digital media platforms. Websites, apps, and social media were the most frequently used channels. Among the studies that used single digital media, Facebook (3/19, 16%), apps (3/19, 16%), websites (1/19, 5%), YouTube (1/19, 5%), and software (1/19, 5%) were used. Multiple devices, including laptops, tablets, and smartphones, were used in 53% (10/19) of the studies. Of the 19 studies, 2 (11%) used smartphones, and 1 (5%) study each used tablets, cell phones, and a special device developed for the intervention, respectively. Approximately 21% (4/19) of studies did not explicitly report the devices used.

Of the 19 studies, 9 (53%) combined multiple modes of communication for the intervention. SMS text messages, images, and videos were typically used. Studies focusing on a single mode used text messages (3/19, 16%), videos (1/19, 5%), or images (1/19, 5%). Approximately 16% (3/19) of the studies were unclear about the modes of communication.

**Table 1:** Format of digital smoking interventions.

Source	Topic	Digital media	Device	Mode	Theoretical model	Conceptual model
Baskerville et al [17], Canada	Smoking cessation	Website, app, Facebook, and YouTube	Laptop and smartphone	Video, image, and articles	No	No
Davis et al [18], United States	Smoking cessation	YouTube and television	Desktop or laptop, mobile device, and television	Digital video advertisements and television advertisements	No	No
Goldenhersch et al [19], Argentina	Smoking cessation	App and cardboard headset device	Smartphone	Video, audio, and group chat	No	No
Guillory et al [20], United States	Tobacco-free lifestyle	Digital and social media (not reported specifically), radio, print, out of home, and local events	Laptop and smartphone	Unclear	Yes	No
Hair et al [21], United States	Antitobacco social movement	Social media, website, YouTube, and television	Laptop, tablet, and smartphone	Unclear	No	No
Kenfield et al [22], United States	Smoking as part of multiple themes	Website, Fitbit One, and SMS text messaging	Laptop, smartphone, and FitBit	SMS text message	No	No
Kim et al [23], United States	Smoking cessation	Website (video, mobile, and search advertisements that direct the campaign website) and display	Laptop, tablet, and smartphone	Video and image	No	No
Kim et al [24], United States	Smoking cessation and reduction	Facebook	Laptop and smartphone	Video-, text- and, image-based materials	No	No
An et al [25], United States	Smoking as part of multiple themes	Websites	Laptop and phone	SMS text message, image, video, and phone call	Yes	No
Marler et al [26], United States	Smoking cessation and reduction	Apps	Other (The Pivot Breath Sensor, a mobile Pivot app)	SMS text message	Yes	No
Masaki et al [27], Japan	Smoking cessation	App, a connected cloud system, a paired mobile exhaled carbon monoxide checker device, and a web-based PC software	Smartphone and laptop	Digital diary, videos, chatbot, and biomedical recording	No	No
Namkoong et al [28], United States	Antitobacco social movement	Facebook	Not reported	Videos, text, and pictures	Yes	Yes
Onezi et al [29], Saudi Arabia	Smoking cessation (smoking relapse prevention)	Twitter and WhatsApp	Not reported	Other (social media–based support groups)	No	No
Romer et al [30], United States	Reduction of influence of peer smoking	YouTube	Not reported	Video	No	No
Thrul and Ramo [31], United States	Smoking cessation	Facebook	Laptop, tablet, and smartphone	Unclear	Yes	No
Tsoh et al [32], United States	Smoking cessation	Facebook	Tablet	Assessment, video messages, 1-page summary printout, or email	No	No
Webb et al [33], United Kingdom	Smoking cessation	App	Smartphone	Video, audio, quizzes, and quit coach through digital chat	No	No
Bary-Weisberg et al [34], Israel	Smoking cessation	Website and SMS text messaging	Cell phone (for SMS text messaging)	SMS text message	No	No
Burford et al [35], Australia	Smoking cessation	Other (The APRIL Face Aging software)	Not reported	Image	Yes	No

Approximately 68% (13/19) of the articles stated that specific theoretical models applied to interventions, whereas 32% (6/19) did not report any models. These models were defined as previously published theoretical models related to social, cognitive, and behavioral factors. Specific models mentioned in the literature include the social cognitive theory [36], transtheoretical models [37], theory of reasoned action, d theory of planned behavior, self-determination theory [38,39], and social branding framework. This study also reviewed the application of conceptual models defined as frameworks designed for specific interventions in the literature. Approximately 95% (18/19) of the studies did not mention any conceptual model specific to the interventions.

### Measures of Dose and Response

The measures used to assess the dose (exposures), response (outcomes), and form of measurement are summarized in Table 2. For response measures, 32% (6/19) of studies reported multiple behavioral outcomes. Measures included smoking status, smoking reduction, abstinence, quit attempts, successful quitting, information search related to smoking cessation, campaign-related topics, and the use of cessation aids. Of the 19 studies, 5 (23%) used multiple psychosocial measures, and 4 (21%) reported a single psychosocial measure. Approximately 37% (7/19) of studies did not report any psychosocial measures. Intention (4/19, 21%), self-efficacy (4/19, 21%), awareness (3/19, 16%), norms (2/19, 11%), and stage of change (2/19, 11%) were mainly used.

For dose measurements, the frequency of exposure, length of exposure, and engagement were assessed. This was designed under the assumption that the amount of dose can be expressed as the sum of the intended dose and the enacted dose; the intended dose equals the frequency of exposure multiplied by the length of exposure planned by the intervention side, and the enacted dose equals the amount of engagement that was actively received by the targeted. Approximately 37% (7/19) of studies explicitly reported the frequency of exposure, whereas 63% (12/19) of studies were unclear on that point. Of the 19 studies, 3 (16%) studies reported the frequency per week, 3 (16%) other studies reported the frequency per day, and 2 (11%) studies reported that the frequency varied for each week. The length of exposure was explicitly reported in 21% (4/19) of studies, whereas 79% (15/19) of studies were unclear about this. All 4 studies that mentioned the length of exposure used videos for the intervention. Engagement was measured in 58% (11/19) of studies. Of the 19 studies, 8 (42%) reported multiple measures, and 3 (16%) reported a single measure. Visits, clicks, log-ins, and views were measured most frequently across different modes of the intervention. Social media interventions reported subscriptions, likes, comments, and postings of content. Finally, 42% (8/19) of studies did not report on engagement.

The dose-response relationship was assessed in 16% (3/19) of studies using different levels of exposure. One of the studies compared the effects of intervention between a standard-dose group and a higher-dose group. Another study examined the interaction between the time of exposure and the treatment arm. One of the studies examined the relationship between participants’ levels of active engagement and the targeted behavior. Outcome assessment was self-reported in 68% (13/19) of studies and a combination of self-report and objective measurements in 21% (4/19) of studies. One of the studies applied only objective measurements using tracking software, and another study used an aggregated self-report measure that assessed population-level awareness.

**Table 2:** Measures used to assess dose and response.

Source	Response (outcome)			Dose (exposure)				Dose-response		Outcome report
	Behavioral	Psychosocial	Frequency of exposure		Length of exposure	Engagement				
Baskerville et al [17], Canada	Smoking cessation and use of cessation aid	Intention to quit	No		No	Visit, installation of the app, and posting content	No		Self-reported	
Davis et al [18], United States	Not measured	Advertisement awareness	No (gross rating points used); depends on the size of the market		Yes (30-second advertisements)	Not measured	Yes		Self-reported	
Goldenhersch et al [19], Argentina	Abstinence	Readiness to quit	Yes (every day)		Yes (10-minute videos)	Not measured	No		Self-reported	
Guillory et al [20], United States	Not measured	Awareness and receptivity	No		No	Not measured	Yes (time×treatment interaction)		Self-reported	
Hair et al [21], United States	Current cigarette use	Advertisement awareness and intentions	No		No	View	No		Other; aggregated self-reported advertisement recall across people grouped by time (weeks) to form a measure of advertisement awareness	
Kenfield et al [22], United States	Smoking	Not measured	Yes (4-5 SMS text messages each week)		No	Activity data from Fitbit, response to SMS text messages, website log-in, and page view	No		Self-reported	
Kim et al [23], United States	Campaign-related topics search	Not measured	No		No	Visit, impressions, and clicks	No		Measured objectively	
Kim et al [24], United States	Smoking reduction	Antismoking attitudes, readiness to quit, motivation to quit, self-efficacy beliefs, and perceived social support	Yes (different each week)		No	Likes and comments	Yes		Self-reported	
An et al [25], United States	30-day abstinence from cigarette smoking	Not measured	Yes (weekly)		No	Not measured	No		Self-reported	
Marler et al [26], United States [26]	Quit attempts, cigarettes per day reduction, and abstinence	Stage of change, desire to quit, readiness to quit, confidence to quit, difficulty to quit, and goals	Yes (>4 times use a day); up to twice weekly SMS text messages		No	Not measured	No		Both	
Masaki et al [27], Japan	Smoking cessation	Not measured	No		No	Not measured	No		Both	
Namkoong et al [28], United States	Smoking-related information seeking	Attitude, descriptive norms, subjective norms, behavioral control, and behavioral intention	Yes (every day)		No	Likes and comments	No		Self-reported	
Onezi et al [29], Saudi Arabia	Smoking cessation and smoking frequency	Not measured	No		No	Subscription to a social media support group	No		Self-reported	
Romer et al [30], United States	Not measured	Smoking norms, mortality beliefs, and smoking attitudes	No		Yes (approximately 4-5 seconds and display of messages)	Not measured	No		Self-reported	
Thrul and Ramo [31], United States	Purposeful 24-hour smoking quit attempt	Not measured	No		No	Not measured	No		Self-reported	
Tsoh et al [32], United States	Smoking abstinence, 24-hour quit attempts, and quit methods	Not measured	No		Yes (videos ranged from 8 to 65 seconds, averaging 29 seconds in length); patients watched 14 to 22 video segments depending on their responses	Session completion and patient-provider discussion	No		Both	
Webb et al [33], United Kingdom	Smoking status, self-reported 7-day point prevalence abstinence at 4 weeks after the quit date, 14-day point prevalence abstinence, and any additional quit attempts after the quit date	Attitudes and perceptions of smoking; self-reported changes in confidence levels, knowledge, attitudes, and perceptions related to smoking cessation; and changes in Smoking Abstinence Self-efficacy questionnaire	No		No	App opens, stage progression, number of messages sent, check-ins, and diary entries	No		Self-reported	
Bary-Weisberg et al [34], Israel	Smoking status	Self-efficacy	Yes (different each week)		No	Keywords sent on text	No		Self-reported	
Burford et al [35], Australia	Successful quitting and quit attempts	Progression along the transtheoretical stages of change model and self-perceptions and attitudes toward smoking behavior	No		No	Not measured	No		Both	

### Quality of Reporting

The clarity of reporting was assessed for media role, dose and response measurement, and funding sources. The findings on clarity of reporting, study design, and bias are summarized in Table 3.

The role of media was clearly reported in all the studies (19/19, 100%). Behavior was clearly measured in all 15 studies that assessed behavioral outcomes. Among the 12 studies that assessed psychosocial outcomes, 11 (92%) reported them clearly, whereas 1 (8%) was unclear about its measurement.

**Table 3:** Clarity of measures and reporting.

Source	Clarity of media role	Response (outcomes)			Dose (exposure)				Study design		Bias		Funding source
		Behavior report	Psychosocial report	Intervention duration		Data collection duration	Engagement report						
Baskerville et al [17], Canada	Yes	Yes	Yes	Unclear		2 to <3 months	Yes	Quasi-experimental study		Generalizability and self-report		Yes	
Davis et al [18], United States	Yes	N/A^a^	Yes	Unclear		Unclear	N/A	Nonexperimental study		Randomization and self-report		Yes	
Goldenhersch et al [19], Argentina	Yes	Yes	Yes	2 weeks to <1 month		2 to <3 months	Yes	Experimental study		Short period of data collection		Yes	
Guillory et al [20], United States	Yes	N/A	Yes	Unclear		>1 year	N/A	Quasi-experimental study		Aided awareness report (recall bias)		Yes	
Hair et al [21], United States	Yes	Yes	Yes	Unclear		>1 year	No	Nonexperimental study		Representativeness		Yes	
Kenfield et al [22], United States	Yes	Yes	N/A	2 to <3 months		2 to <3 months	Yes	Experimental study		Representativeness		Yes	
Kim et al [23], United States	Yes	Yes	N/A	2 to <3 months		2 to <3 months	Yes	Nonexperimental study		Generalizability		Yes	
Kim et al [24], United States	Yes	Yes	Yes	2 weeks to <1 month		1 to <2 months	Yes	Nonexperimental study		Self-report, small sample size, and representativeness		Yes	
An et al [25], United States	Yes	Yes	N/A	1 to <2 months		2 to <3 months	N/A	Experimental study		Self-report (recall bias)		Yes	
Marler et al [26], United States	Yes	Yes	Yes	2 to <3 months		2 to <3 months	N/A	Nonexperimental study		Generalizability		Yes	
Masaki et al [27], Japan	Yes	Yes	N/A	4 to <6 months		6 months to <1 year	N/A	Experimental study		Representativeness		Yes	
Namkoong et al [28], United States	Yes	N/A	Yes	2 weeks to <1 month		2 weeks to <1 month	No	Quasi-experimental study		Representativeness		No	
Onezi et al [29], Saudi Arabia	Yes	Yes	N/A	Not reported		Not reported	Yes	Nonexperimental study		Generalizability and cross-sectional		Yes	
Romer et al [30], United States	Yes	N/A	Yes	Not reported		Not reported	N/A	Quasi-experimental study		Generalizability and control setting		Yes	
Thrul and Ramo [31], United States	Yes	Yes	N/A	1 to <2 months		6 months to <1 year	N/A	Nonexperimental study		Self-report (recall bias), representativeness, and low test power		Yes	
Tsoh et al [32], United States	Yes	Yes	N/A	Other		2 to <3 months	Yes	Nonexperimental study		No control and self-report		Yes	
Webb et al [33], United Kingdom	Yes	Yes	Yes	2 weeks to <1 month		6 months to <1 year	Yes	Experimental study		Generalizability and biased sample		Yes	
Bary-Weisberg et al [34], Israel	Yes	Yes	Yes	4 to <6 months		Unclear	Yes	Nonexperimental study		Representativeness		Yes	
Burford et al [35], Australia	Yes	Yes	No	Not reported		4 to <6 months	N/A	Experimental study		No blinding		No	

^a^N/A: not applicable.

The duration of intervention was unclear in 21% (4/19) of studies and was not reported in 16% (3/19) of studies. Of the 19 studies, 12 (63%) reported this explicitly. The intervention duration ranged from 10 minutes to 6 months. One of the studies (1/12, 8%) reported it in the range as it varied among participants because of the customization function of the digital intervention. Of the 19 studies, the intervention duration was 2 weeks to a month in 4 (33%) studies, 1 to 2 months in 2 (17%) studies, 2 to 3 months in 3 (25%) studies, and 4 to 6 months in 2 (17%) studies. The duration of data collection was unclear in 11% (2/19) of studies and was not reported in 11% (2/19) of studies. It ranged from 2 to 3 months in 47% (7/15) of studies, 6 months to a year in 20% (3/15) of studies, and >1 year in 13% (2/15) of studies. Approximately 7% (1/15) of studies each collected data for 2 weeks to a month, 1 to 2 months, and 4 to 6 months, respectively. The reporting of engagement was clear in 82% (9/11) of the studies that assessed engagement.

Of the 19 studies, 6 (32%) applied experimental study designs, 4 (21%) used quasi-experimental designs, and 9 (47%) used nonexperimental study designs. Quasi-experimental designs were defined as studies with a control group that did not involve random assignment. Of the 6 experimental studies, 3 (50%) reported representativeness and a biased sample as a potential source of bias. Quasi-experimental studies reported that self-reporting and aided recall were threats to bias (2/4, 50%). Lack of randomization, self-reporting, small sample size, and lower statistical power were frequently reported, in addition to generalizability and representativeness among nonexperimental studies. Of the 19 studies, 17 (89%) reported a source of funding, and 2 (11%) did not report this information.

## Discussion

### Principal Findings

This study identified the literature on digital behavior change interventions related to smoking and addressed the current practice of measuring and reporting intervention contents, channels, and dose and response outcomes. Data synthesis showed that both measurement and reporting were inconsistent across studies, illustrating the current challenges in this field of research.

This review revealed a lack of preventive studies on tobacco and nicotine use. Among 324 digital behavior change intervention papers, only 19 (5.9%) papers were relevant to smoking, and none centered on preventing its initiation. Although a literature search detected numerous prevention research papers, this review only included papers that assessed behavioral and psychosocial outcomes and did not include studies that focused on perceptions and engagement. This lack of research may reflect the fact that prevention studies for digital interventions remain at an early stage of identifying the basic conditions to make interventions engaging and effective rather than measuring the effectiveness of such interventions in achieving behavior change. This may also be because of a lack of funding in prevention research, as well as challenges in long-term follow-up to detect differences in smoking initiation rates.

Another gap in the literature on digital health identified in this review was the dose-response relationship. Although the effectiveness of interventions depends on the amount of exposure, only 16% of the literature has assessed the outcomes across different levels of doses.

The biggest issue was the inconsistency and vagueness of the reporting. Most studies paid attention to reporting outcomes and were relatively clear on their measurements in the methods sections. However, reporting of the amount of intervention offered to participants and the actual engagement was often unclear and not explicitly mentioned in a large proportion of studies. Insufficient details on the amount of exposure make it more difficult to compare outcomes across studies and conduct meta-analyses, which hinders the provision of evidence on effective digital interventions.

These findings were consistent with previous research on digital health interventions. Hu et al [3] found that only 10.6% of papers on digital health focused on health promotion and interventions, and only 2.6% centered on substance use, including tobacco use. Abad et al [4] pointed out that only 6.8% of the literature on digital health focused on smoking. Another review by Seiler et al [1] demonstrated that there have been relatively few rigorous studies on the effectiveness of digital behavior change interventions, and the reporting of design, measures, data collection, and other methods need to be improved [5].

The amount of exposure to an intervention can be divided into the intended dose planned by the intervention side and the enacted dose that was completed by the participant side. Dose can be expressed as the frequency multiplied by the length of the intended intervention dosage, and the amount of engagement can offer information about the enacted dosage. Clarifying these elements in the literature will lead to the promotion of comparable reporting and advancement of the evidence development of digital health interventions.

The types of behaviors used to report the outcomes were mixed. It can be divided into (1) targeted behavior (ie, smoking cessation), (2) surrogate behavior (ie, smoking reduction), and (3) behavior related to the improvement of the likelihood of conducting targeted behaviors (ie, use of cessation aid and smoking cessation information search). Although the measurement of these outcomes was predominantly self-reported, some studies combined objective measurements. The methods of objective measurement included (1) biochemical devices (eg, exhaled carbon monoxide tracker), (2) automatic digital tracking (eg, for information searches), (3) electronic health records, and (4) group-level psychological measures (eg, group-level awareness of campaigns). Leveraging these emerging methods will enhance the validity and reliability of measurements and advance evidence-based digital health interventions.

### Strengths

To the best of our knowledge, this is the first study to review the dose and response measurements of digital smoking interventions. Dose measures were divided into the intended dose from the intervention side and the enacted dose that was actually completed by the participant side. The intended dose was separately assessed for frequency and length. Response measures were organized into behavioral and psychosocial outcomes, and measurement methods were assessed to determine their validity. The proportion of dose-response relationship studies was identified to determine the stage of current research in the field. The literature search combined general and journal-specific searches and yielded 2916 studies that showed high coverage.

Research gaps exist in assessing digital behavior change interventions with clear dose and response measurements and the dose-response relationship between the levels of intervention exposure and outcomes. More prospective studies are needed to examine the relationship between higher and lower dosages of interventions on smoking outcomes. For example, a well-designed dose-response relationship study on vaping outcomes among a specific population will identify the effective amount of digital intervention to prevent the initiation and decrease the amount of e-cigarette use. This will provide evidence for identifying the effective amount of intervention and offer grounds for conducting well-designed meta-analyses that synthesize evidence for digital behavior change interventions. This paper contributes to building a base for these studies.

### Limitations

This review had several limitations. First, the number of studies included in this analysis was small. This was mainly because of the lack of digital behavior change intervention studies related to smoking and the measurement of behavioral and psychosocial outcomes in the current phase in the area. Second, the risk of bias assessment was omitted from the review. This was because of the nature of the mixed study designs in the included studies as there is little risk of bias assessment tools intended to apply to a variety of study designs. Instead, the quality of the papers was assessed by reviewing and evaluating the study design, theoretical model application, conceptual model application, cost, funding source, and bias source. In addition, the clarity of the media role and every measure of dose and response were assessed as part of the review for each study. When digital smoking behavior change interventions become more common and more literature can be included in the future, these limitations can be minimized.

### Conclusions

There are challenges in every emerging area of scientific research. Digital smoking interventions are a new and growing area of research involving continuous and rapid advancements in technologies for health intervention delivery, implementation, and dissemination. This characteristic implies that it is essential to use consistent, standardized methods to evaluate the outcomes to accurately understand the efficacy and effectiveness of the interventions in rapid change, particularly in this area. For future improvement in this realm, this study assessed the consistency of measures used to quantify the dose and response in digital smoking interventions. These results suggest that both dose and response measures are often not clearly defined and are inconsistent across studies. This can imply that synthesizing the evidence on digital smoking interventions has been a challenge, and past studies could have been based on vague reporting and outcome evaluations. This is particularly an issue as interventions to date could be based on biased findings.

This study details and structures the composition of evaluation measures and assesses their use in digital smoking intervention studies. To the best of our knowledge, this is the first study to review dose and response measurements of digital smoking interventions. Although it has limitations such as the small number of papers included and the limited risk of bias assessment, the study successfully (1) reviewed and codified measures used for digital health interventions in tobacco and nicotine use research, (2) evaluated the quality of reporting of dose and response, and (3) identified areas for improvement in the field. More prospective studies that examine the clarity and consistency of measures among a larger number of studies are needed to develop further grounds for evidence-based digital smoking interventions. There is also a need for a clear and consistent reporting scheme for digital health interventions to accurately evaluate outcomes and conduct well-designed meta-analyses. Provision of clear and consistent reporting of both outcomes and exposures is needed to develop further evidence in this field that leads to protecting the lives and health of the public.

## Abbreviations

PRISMA: Preferred Reporting Items for Systematic Reviews and Meta-Analyses
PROSPERO: International Prospective Register of Systematic Reviews
